# A randomized trial of a theory-driven model of health coaching for older adults: short-term and sustained outcomes

**DOI:** 10.1186/s12875-023-02162-x

**Published:** 2023-10-05

**Authors:** Kathleen Potempa, Margaret Calarco, Marna Flaherty-Robb, Susan Butterworth, Deanna Marriott, Stacia Potempa, Candia Laughlin, Patricia Schmidt, Laura Struble, Karen Harden, Bidisha Ghosh, Philip Furspan, Alexis Ellis

**Affiliations:** 1 School of Nursing, University of Michigan, 400 N. Ingalls St., Ann Arbor, MI 48109 USA; 2 The University of Tennessee Health Science Center, 920 Madison Ave., Memphis, TN 38163 USA

**Keywords:** Health coaching, Health behavior, Health surveys, Aging, Chronic disease, Hypertension, Nurses, Videoconferencing, Internet

## Abstract

**Background:**

Healthy Lifetime, a theoretically driven, personalized health coaching program delivered electronically, including face-to-face videoconferencing, was developed to intervene in early aging to stave off functional decline and minimize the onset/exacerbation of chronic conditions.

**Objective:**

To determine the efficacy of a theoretically driven, personalized health coaching program in participants 50 years and older with one or more chronic conditions using a randomized, controlled, pragmatic clinical trial methodology.

**Methods:**

Participants were randomly assigned to the HL (*n* = 59) or a usual care (*n* = 63) group. The HL group received health coaching from a trained nurse over eight weeks. Outcomes were measured at baseline, eight weeks, and 20 weeks (after the 12-week no-treatment phase). Regression modeling with fixed-effect repeated measures was used to account for the longitudinal data collection.

**Results:**

For the HL group, *health habits* increased at 8 weeks (3.1 units; SE = 1.0; *p* = .0005; effect size = .15). This difference was sustained at 20 weeks (2.4 units, SE = 0.2; *p* = .0005). *Independent self-care agency* improved at 8 weeks in individuals with high blood pressure (13.5 units; SE = 4.37; *p* = .0023; effect size = .3). However, that difference was not sustained at 20 weeks (*p* = .47). No significant improvements were shown in the usual care group at 8 weeks or 20 weeks.

**Conclusions:**

HL participants significantly improved their health habits at 8 weeks and sustained this improvement at week 20 (after a 12-week no-treatment phase) vs. the usual care group. Changing health habits alone has been shown to reduce all-cause morbidity and mortality in chronic disease. The high-functioning, community-dwelling older adults with chronic diseases we studied is an important target population for primary care practices to intervene early in aging to stave off the complications of chronic disease and functional decline.

**Trial registration:**

ClinicalTrials.gov (record NCT05070923, 07/10/2021).

**Supplementary Information:**

The online version contains supplementary material available at 10.1186/s12875-023-02162-x.

## Background

A significant and growing proportion of people seen in primary care practices are 50 and over with one or more chronic conditions [1, 2]. In addition to managing the disease process, primary care practices are challenged with health promotion to improve lifestyle habits in this population, as these significantly impact clinical outcomes in chronic diseases [3]. For example, there is abundant literature showing the effect of specific health habits on all-cause mortality and morbidity [3–6], such as inadequate exercise, sleep, poor food choices, use of alcohol, smoking, and the negative impact of socio-behavioral factors such as poor health-related quality of life [7]. Yet, a paucity of evidence-based approaches or programs are available to assist primary care practices in improving lifestyle and socio-behavioral factors.

Health coaching is an often-espoused method to assist adults in changing their lifestyle [8], yet results across randomized controlled trials (RCTs) are highly variable. An integrative review published in 2010 yielded 15 peer-reviewed studies published between 1999–2008. Significant improvements in one or more health behaviors were identified in only six of the 15 studies [40%]. Intervention strategies described in the studies also varied, including goal setting [73%], the use of motivational interviewing [27%], and collaboration with health providers [20%]. In a more recent systematic review of health coaching RCTs [9], only one of six studies cited showed significant between-group effects after 40 weeks of coaching [10]. Three studies cited in this review measured physical activity and showed no significant between-group results [11–13]. In a 2023 systematic review and meta-analysis [14] examining the behavioral change techniques (BCTs) used in health coaching-based intervention for Type 2 Diabetes (T2DM), few BCTs were used in the interventions.

Inadequate, imprecise descriptions of interventions and the need for more theory-based methods were the main limitations of the studies reviewed. Behavior change techniques and coaching protocols need to be transparent and replicable to build the evidence base of effective health coaching interventions [14]. Moreover, recent reviews of health coaching interventions found that telehealth technology improved accessibility, especially for older adults [15], and others have shown older adults' acceptance and facility of teleconferencing [16, 17].

Considering this literature and prior limitations in health coaching interventions, we developed a theoretically driven, nurse health coaching program incorporating specific BCT strategies accessible to a broad group of older adults using the videoconferencing eHealth methods called the Healthy Lifetime program. Our program's overall goal is to intervene in early aging when individuals have the best chance for more extended-term benefits of changing their health behavior, staving off functional decline, and delaying the onset or exacerbation of chronic conditions [18].

The principal aim of this study was to determine the efficacy of the Healthy Lifetime program using a randomized, controlled, pragmatic clinical trial methodology in participants 50 years and older with one or more chronic conditions. This paper provides the findings of this recently completed pragmatic randomized trial. The theoretical framework, BCT strategies, and intervention protocol are previously described [19].

## Methods

A randomized 2 × 3 repeated measures design was used. Survey measurements were taken at three-time points, baseline, at 8 weeks (after the 8-week coaching intervention), and at 20 weeks (after a 12-week no-treatment phase to determine the sustainability of outcomes). We employed a randomization sequence using Excel 2007 (Microsoft, Redmond, WA, USA) with a 1:1 allocation using random block sizes of 2 and 4 by a blinded team member (statistician) to randomize participants to either the Healthy Lifetime (HL) coaching intervention or the usual care group. The study was registered with CT.gov on 09/23/2021 (record NCT05070923) and was approved by the Institutional Review Board (IRB).

### Target population

People 50 years of age and older with one or more chronic health conditions were non-randomly recruited using community outreach methods. The inclusion and exclusion criteria are listed in Table 1. We aimed to recruit 120 individuals who met inclusion criteria and signed the IRB-approved informed consent to participate [60 in each group]. As a pragmatic trial with fixed grant funding for the study, we wanted to ensure that our proposed, efficient sample size had adequate power to detect small effects [20]. Our sample size calculation was based on our regression model that accounts for using all three time points in a single analysis. Using this model at a 5% alpha-level test, our proposed sample size of 120 participants has an 80% power to detect an effect size of 0.07, considered a small effect.

**Table 1:** Inclusion and exclusion recruitment criteria

**Inclusion Criteria**	**Exclusion Criteria**
50 years of age or older who have one or more chronic medical conditions (e.g., high blood pressure, diabetes, arthritis, obesity, etc.) which require management in some way (regular doctor checks, medication, etc.);	Are acutely ill or have unstable health problems requiring medical work-up or follow-up clinic visits for monitoring more than every 3 months;
Whose health is medically stable, that is, not currently undergoing either significant physical and/or mental health changes and not undergoing any type of non-routine treatments/medical testing or have any surgeries scheduled in the next six months;	Have had an ER visit related to his/her chronic condition in the prior one month; (an ER visit related to a *one-time, resolved issue* such as a bee sting or to have stitches for a household injury will not be cause for exclusion);
Has not had an ER visit related to his/her chronic conditions in the prior one month (an ER visit related to a *one-time, resolved issue* such as a bee sting or to have stitches for a household injury will not be cause for exclusion);	Are terminally ill;
Can read, speak, and hear English; may use adaptive devices such as hearing aid and glasses;	Have severe memory problems;
Can recall personal information such as age, DOB, address, phone number, and health history questions without difficulty;	Have severe hearing and/or visual deficits that are not functionally adapted with devices such as a hearing aid or eyeglasses;
Reports having access to a computer and an established internet connection that is regularly used for video content (such as with Netflix, Amazon Prime, and YouTube); and	Do not have a computer or an existing internet connection at the bandwidth needed to support the video platform (cannot access video streaming content); and/or
Can use their internet connection in a private space	Can use internet only in a public space (unable to ensure privacy)

### Recruitment

Participants were recruited using various methods, including direct mailings of the study description and flyers to individuals on the 2018 list of registered voters in Michigan and member lists of senior centers throughout Michigan. To encourage gender, racial/ethnic, and economic diversity, we included direct mailings to Medicare and Medicare/Medicaid dual-eligible beneficiaries from the Michigan Department of Health and Human Services and to the Healthier Black Elders Center’s (HBEC) [21] registry in Detroit, Michigan, which is part of the Michigan Center for Urban African American Aging Research. The study coordinator managed participant recruitment. Once a participant was screened and enrolled, another team member (project manager) assigned the participant to the group aligned with the randomization scheme. Because the intervention was delivered online, participants were blind to other participants. The study was conducted in a large Midwestern catchment area from October 2020 through December 2021.

### Treatment groups

**Fig. 1:**
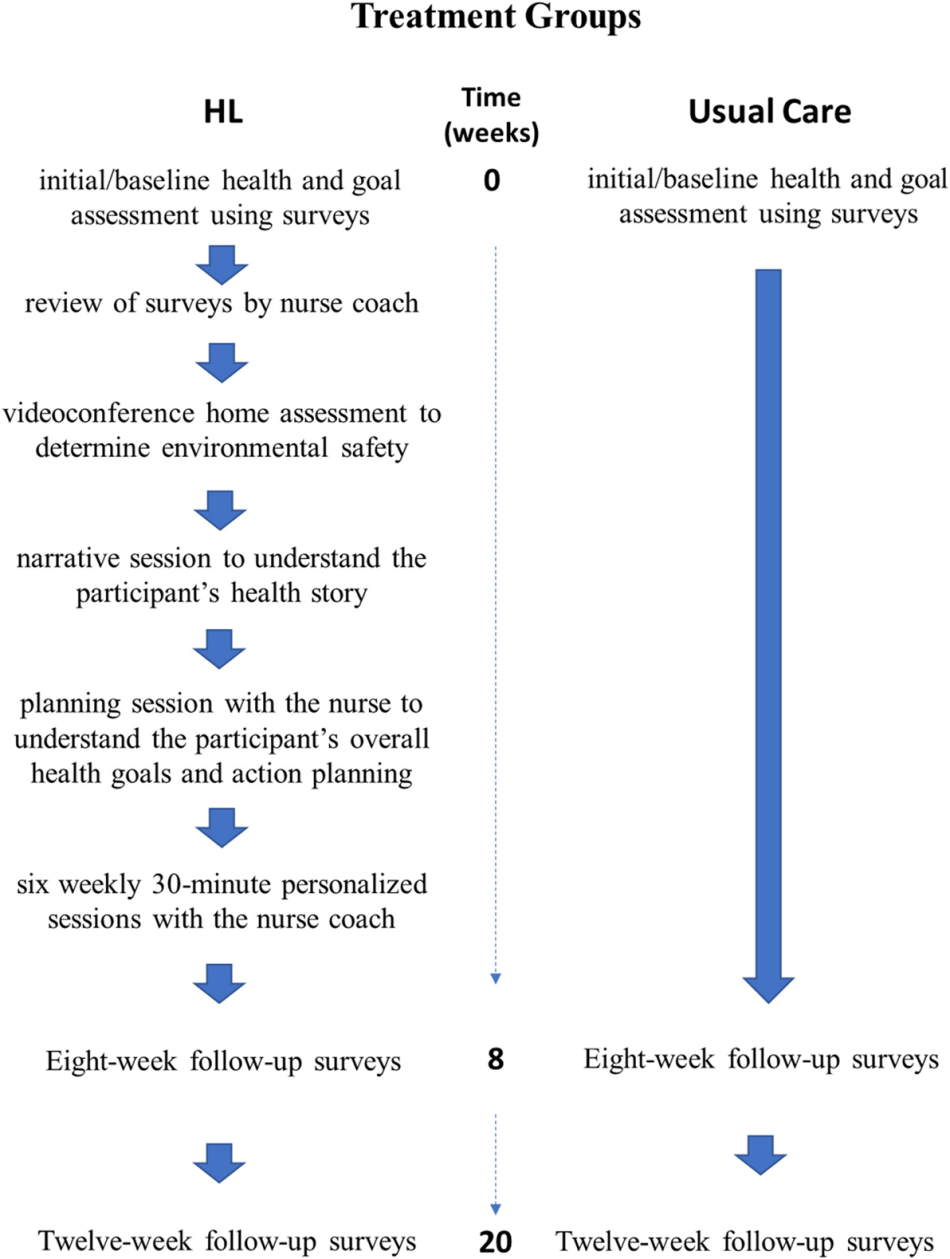
Treatment process for Healthy Lifetime and Usual Care groups

The intervention phase of the study was eight weeks, followed by a 12-week no-treatment phase. Figure 1 shows the protocol flow for the HL and usual care groups. A secure website (gethealthie.com) was used to conduct the study for all participants. Survey information, outcome assessments, and all nurse-participant coaching interactions were delivered/conducted using two-way videoconferencing (Zoom.com) via the study website (gethealthie.com). The HL intervention sessions with participants included the following: (1) an initial health and goal assessment survey completed online; (2) a review of the surveys by the nurse coach; (3) a two-way videoconference home assessment to determine environmental safety; (4) a narrative session to understand the participant’s health story; (5) a planning session with the nurse to understand the participant’s overall health goals and action planning; and (6) six weekly 30-min personalized sessions with the nurse coach.

The HL 8-week intervention focused on person-centered engagement, client empowerment, and cognitive-behavioral and narrative coaching approaches, with the overall communication approach based on motivational interviewing (MI) [22–24]. As previously reported, [25] specific BCT strategies were individualized and tailored to address each participant's unique driving and restraining forces for behavior change. The coaching approach provided a blend and balance of coaching strategies to build self-care capacity and improved functioning over the intervention [19, 22, 23, 26–28]. Nurse coaches were blind to all participants other than their assigned individuals. Participant satisfaction with the HL program was determined at the end of the 8-week intervention by administering a five-question survey using a 6-point scale ranging from 0-Not at all satisfied to 5-Highly satisfied.

The usual care group received instructions from the study coordinator when it was time to fill out their health surveys at baseline, at 8 and 20 weeks. “Usual care” is defined as the ongoing activities of medical surveillance (such as medical provider visits), case management if needed (such as with Medicare/Medicaid dual eligible care management program), emergent care if needed (such as hospitalization), or other routine health activities such as health club membership. No continued contact with the “usual care” group occurred.

The HL intervention group and the “usual care” group received the 99-item Personal Health Survey (PHS; Appendix), which included health-related questions enabling all participants to reflect on their current health status and lifestyle behaviors. In addition, the PHS asks the participant to identify up to three health goals they may be working toward, such as weight loss, exercise, etc. As such, the HL intervention participants and “usual care” group participants may benefit from focusing on these questions as part of the study process.

### Measures

The participants completed the PHS self-report survey three times: at baseline before randomization, at eight weeks, i.e., at the end of the intervention phase, and 20 weeks – twelve weeks after the end of the intervention no-treatment follow-up phase. The survey was used for several purposes: to focus participants on their health and health-related goals, to provide participants’ health background for the nurse coaches, to describe the sample, and to use some measures as independent or dependent variables in the analyses. Unless otherwise indicated, individual and composite scales that comprised the PHS survey were taken from the Self-Management Resource Center of Stanford University (SMRC) [29] with previously reported psychometric properties. We could not include biometric measures such as body weight, blood pressure, etc., because the study occurred during the lockdown phase of the COVID-19 pandemic when most participants were in quarantine and could not obtain in-home or clinic measurements from a health professional.

Table 2 displays the demographic and other descriptors as well as the outcome measures of the study. The descriptive variable *Level of Independence in Activities* is the Instrumental Activities of Daily Living (IADL) checklist of the Senior Planning Services, Santa Barbara, California (used with permission) [30] with a 5-point Likert scale that ranges from 1 “Cannot Do” to 5 “Do independently.” Outcome measures included level of exercise/activity (including stretching and aerobic activity items specific for individuals with chronic conditions (SMRC)) and eating habits (positive and negative food choices that included major food group recommendations of the 2015–2020 *Dietary Guidelines for Americans*) [31]. A total score for the latter measure, where higher scores represent better or worse choices, included *Positive Food Choices* ***,*** which represent groups recommended to be added for a healthier diet, and Negative Food Choices, which represent items recommended to be reduced for a more nutritious diet. Use of alcohol included the number of total mixed drinks, beers, and glasses of wine consumed in a week with an 8-point scale ranging from less than one/week to greater than eight drinks/week. We calculated a composite *health habits* score (defined as hours of *exercise/activity per week* + *Positive Food Choices* (e.g., fruits and vegetables) – *Negative Food Choices* (e.g., fats and sugar), and *number of alcoholic drinks per week*) as both the intervention and the “usual care” group reported two or more top priority health goals related to everyday public health messaging regarding risk reduction, i.e., body weight, food choice, use of alcohol, smoking, and exercise [32]. Smoking was not included in our composite because few participants reported smoking. Additional measures were *level of functional impairment* (defined as the impact of their chronic disease on health and the level of symptomatology experienced), *independent self-care agency* (defined as the confidence in maintaining independent functioning across a range of activities such as housekeeping, transportation, errands/chores as well as confidence in managing aspects of chronic disease), *confidence in achieving health-related goals* (defined as the self-identified priority goals put forth at the baseline of the study), and *self-efficacy toward medication-taking* (defined as confidence in managing their medication taking across multiple circumstances, e.g., when on vacation or if ill).

**Table 2:** All study measures (PHS Survey Items)

**Demographic**
i. Year of Birth [Q1]
ii. Gender [Q2]
iii. Marital status [Q3]
iv. Race/ethnicity [Q4]
v. Level of education [Q5]
vi. Work status [Q6]
vii. Household income [Q7]
**Baseline Covariates/Moderators**
Social Network/Support [Q8-Q14]
Number of Prescribed Medications (Taken from Medical History/Medications not PHS)
Level of Independence IADL [Q53-Q60]
Major Categories of Chronic Diseases (Taken from Medical History/Medications not PHS)
**Outcomes**
1. Level of exercise/Activity [Q27-Q31; higher score is more exercise/week]
2. Eating and Drinking (composite measure = Total Score of positive food choices[Q32-Q34] – Total Score of Negative food choices [Q35 and Q40] – Total Number of Alcoholic drinks/week – if no drinks 0, if < 1 = 1. Higher is better food/drink
3. Health Habits That Modify Risk of Chronic Disease: Total Score exercise/activity + Total score positive food choices – total score negative food choices – Total number of Alcoholic drinks/week. Higher score is better health habits
4. Functional Impairment: [Total Score of Q41-Q44 *impact of health* + Total score of Q45-50 *symptoms*]; higher value is more impairment
5. Independent Self Care Agency [Q61 – Q73]. Higher value is higher agency
6. Overall confidence in Achieving Goals Selected: Compute the average confidence score across available scores: Q92 + Q95 + Q98/3 or the # scores available;
7. Goal Setting Competency: Overall importance of goals selected + overall confidence in achieving goals selected (higher is better)

### Statistical analysis

Descriptive statistics are reported using means and standard deviations for continuous variables and frequencies and percentages for categorical variables. To assess the impact of the HL intervention on participant outcomes, we performed regression modeling with fixed-effect repeated measures to account for the longitudinal data collection at baseline, eight weeks, and 20 weeks. We used logistic regression for dichotomous outcomes and linear models with transformations as needed for continuous outcomes. The candidate variables for inclusion in the model are the demographic, descriptive, and outcome variables listed in Table 2. Covariates included age, race, gender, education level of income, and whether the participant had a partner. The primary variable of interest is the interaction between group and time. The parameter associated with this interaction indicates a change in trajectory over time between the HL group and the “usual care” group. Also, two candidate effect modifiers – the number of medications taken, and high blood pressure (HBP) were included in a three-way interaction with group and time due to their potential to modify the effectiveness of the intervention. Hypertension was conceptualized as a measure of medical risk as it represented the dominant chronic disease. Health behavior plays a significant role in preventing and treating hypertension in our target population.

To prevent over-fitting and the small sample size relative to many predictors, we used a forward selection technique at an alpha level of 0.05. This approach allowed us to identify a parsimonious set of variables independently associated with the outcome variables. We examined residual, quantile, and leverage plots to test the model's fit. Trajectories over time and interactions are reported. The statistical package used was SAS 9.4 (SAS Institute, Inc., Cary, NC). The statistician and data manager, uninvolved in protocol management, conducted data verification and analyses.

## Results

### Sample

One hundred thirty-one participants were recruited. A final sample of 122 that completed screening and baseline surveys was randomized into the HL (59 participants) or “usual care” groups (63 participants). One hundred sixteen participants completed surveys at all three time points: baseline, after eight weeks, and at 20 weeks. For these 116 participants, missing data was minuscule; missing data cells were dropped from the analysis. All randomized participants were included in the analyses.

Table 3 displays these participants' demographic and descriptive characteristics overall and stratified by intervention versus the “usual care” group. The *level of Independence in Activities* mean (SD) was 37.65 (5.24) out of a maximum possible score of 40, very high for older adults with one or more chronic diseases [33]. Because of this ceiling effect, it was not included as a predictor variable. The *Social network and support* variable had limited variability and was excluded from analyses. The *top priority goals for* the HL and “usual care” groups were diet/weight, activity/exercise, and physical health. Participants ranged from 51 to 93 years of age, with a mean (SD) of 71 [9]. Almost 82% of the participants were female, and 40% lived with a spouse or a partner. 52% of the sample was African American or other non-White races, and 48% were White.

**Table 3:** Demographic and descriptive characteristics of participants

	Overall	HL	“Usual care”
	*N* = 122	*N* = 59	*N* = 63
**Categorical Variables n (%)**			
Sex			
Female, n (%)	99(81.8)	48(48.5)	51(51.5)
Male, n (%)	21(17.4)	11(52.4)	10(47.6)
Prefer not to answer, n (%)	1(0.8)	0(0)	1(100)
Living with spouse/Partner			
Yes, n (%)	48(40.0)	21(43.7)	27(56.3)
No, n (%)	72(60.0)	38(52.7)	34(47.2)
Education			
Less than Bachelor's, n (%)	43(35.8)	23(53.5)	20(46.5)
Bachelor's, n (%)	32(26.7)	17(53.1)	15(46.9)
Graduate/Professional, n (%)	45(37.5)	19(42.2)	26(57.8)
Race			
African American, n (%)	54(45.0)	29 (49.2)	25(41.0)
White, n (%)	58(48.3)	26(44.1)	32(52.5)
Other, n (%)	8(6.7)	4(6.8)	4(6.6)
Prefer not to answer, n (%)	2(1.6)	0(0)	2(3.2)
Income group			
$0—$20,000, n (%)	22(18.4)	12(54.5)	10(45.5)
$21,000—$50,000, n (%)	40(33.6)	19(47.5)	21(52.5)
$51,000—$100,000, n (%)	41(34.5)	24(58.5)	17(41.5)
Greater than $100,000, n (%)	16(13.5)	3(18.75)	13(81.25)
Number of participants with chronic conditions/diseases:			
Chest pain	7	5	2
Slow heartbeat	6	5	1
Fast heartbeat/palpitations	14	6	8
High blood pressure	65	30	35
Swelling in legs	17	11	6
Cold hands/feet	30	12	18
Heart murmurs	14	10	4
Heart attack	4	4	0
History of rheumatic fever	0	0	0
Diabetes	17	9	8
**Continuous Items: Mean (SD)**			
Age, mean (SD)	71.0(9.0)	72.7(9.0)	69.4(8.8)
Number of medications, mean (SD)	5.0(3.0)	5.2(2.9)	4.8(3.1)
Baseline Priority Health Related Goals by Category [Q91,94,97], # of mentions			
Exercise/Activity	75	34	41
Diet/Weight	88	45	43
Health – Physical	69	31	38
Health – Mental	22	10	12
Sleep	15	8	7
Medication	6	2	4

Regarding education, 36% had less than a bachelor’s degree, approximately 27% had a bachelor’s degree, and 37% had a graduate or a professional degree. About 68% of the population belonged to the two middle-income groups, with 34% in the $21 K—$50 K range and 34% in the $51 K—$100 K range. A small minority made less than $20 K (18%) or more than 100 K (14%).

### Evaluation outcomes

**Fig. 2:**
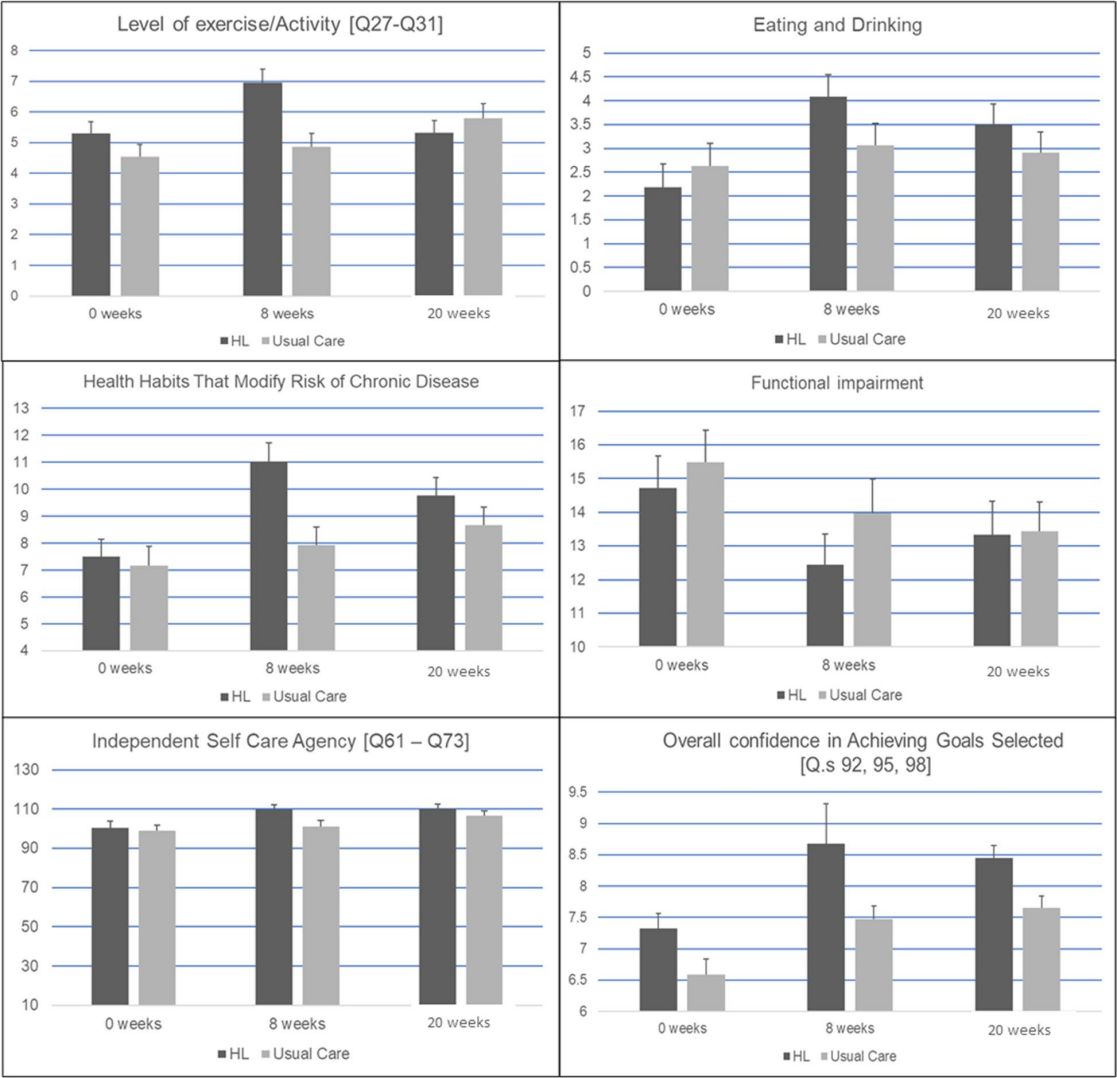
Histograms of outcome variables indicate improvement trends over time for *both* the HL and “usual care” groups

Means (SE) of all outcome variables at baseline, at eight weeks, and 20 weeks are displayed in Table 4. Graphs illustrative of change over time by the group are shown in Fig. 2 for the outcomes of exercise/activity, eating and drinking, health habits, independent self-care agency, level of functional impairment, and goal-setting confidence. Ceiling effects were found for two variables, *goal-setting importance* and *goal-setting competency,* and were not graphed or included in the regression model. The variables in the regression analysis, *health habits,* and *independent self-care agency* showed a significant interaction between group and time. Table 5 displays the regression coefficients for these models.

**Table 4:** Summary statistics for outcome variables

Variable	Group (Mean, SD)		Group (Mean, SD)		Group (Mean, SD)	
	**Time = 0**		**Time = 8**		**Time = 20**	
	HL (*n* = 59)	Usual Care (*n* = 63)	HL (*n* = 58)	Usual Care (*n* = 61)	HL (*n* = 57)	Usual Care (*n* = 59)
Exercise Composite Measure. *Higher is more exercise*	5.31 (2.85)	4.54 (3.25)	6.95 (3.43)	4.87 (3.3)	7.36 (3.85)	5.76 (3.75)
Eating and Drinking—*Higher is better*	2.19 (3.72)	2.63 (3.76)	4.09 (3.51)	3.07 (3.57)	3.61 (3.47)	2.91 (3.38)
Health Habits that modify risk of Chronic Disease—*Higher is better*	7.49 (5.04)	7.17 (5.66)	11.03 (5.22)	7.93 (5.17)	9.75 (5.07)	8.67 (5.07)
Functional Impairment—*Higher value is worse*	14.71 (7.29)	15.49 (7.52)	12.45 (6.86)	13.98 (7.83)	13.22 (7.5)	13.43 (6.76)
Independent self-care agency—*Higher score is better*	100.36 (25.39)	98.98 (21.46)	109.71 (19.54)	101.07 (23.3)	110.53 (19.8)	107.29 (17.98)
Importance to achieve these goals (average)- NUMERIC	8.74 (1.36)	8.49 (2.23)	9.1 (1.57)	8.6 (1.81)	9.01 (1.26)	8.83 (1.07)
Confidence to achieve these goals (average)- NUMERIC	7.26 (1.93)	6.6 (1.92)	8.68 (1.81)	7.47 (1.69)	8.47 (1.48)	7.65 (1.5)
Goal setting competency—*Higher is better*	16.0 (2.77)	15.37 (3.0)	17.77 (3.15)	16.33 (2.61)	17.56 (2.37)	16.49 (2.23)
Q74-85 Medication taking self-care efficacy. *Higher score is more confident*	20.61 (3.36)	19.57 (4.72)	22.78 (2.09)	20.87 (4.7)	21.86 (2.77)	20.87 (4.0)

**Table 5:** Multivariate regression analyses with fixed-effects repeated measures

		Multivariable Analysis		
		Coefficient	Standard Error	*p*-value
**Outcome 1: Health Habits that modify risk of Chronic disease**				
Intercept (Multivariable)		6.7	0.95	
Time	0	(reference)		.0011
	8	0.72	0.85	
	20	1.28	0.86	
Interaction: Time and Group	Time*Group			.0005
		(reference)		
	Time = 8*Group = HL	3.06	1	
	Time = 20*Group = HL	2.35	1	
Income	Greater than $100,000	(reference)		.0056
	$0—$20,000	1.33	1.04	
	$21,000—$50,000	3.22	0.93	
	$51,000—$100,000	1.47	0.93	
Number of Medications		-0.22	0.1	.0241
**Outcome 2: Independent Self-Care Agency**				
Intercept (Multivariable)		104.1	3.86	
Time	0	(reference)		.0071
	8	1.83	2.91	
	20	8.13	2.95	
Living with spouse/Partner	Yes	(reference)		.0117
	No	-8.11	2.74	
White	Yes	(reference)		.0037
	No	7.08	2.42	
Education	Graduate/Professional	(reference)		.0147
	Less than Bachelor's	-5.42	2.85	
	Bachelor's	4.69	2.97	
Income group	Greater than $100,000	(reference)		.0042
	$0—$20,000	5.09	4.77	
	$21,000—$50,000	11.27	4.21	
	$51,000—$100,000	4.03	3.62	
Time*Group*High BP		(reference)		.007
	Time = 8*Group = HL *High BP = Yes	13.46	4.37	
	Time = 20*Group = HL *High BP = Yes	3.19	4.4	
Number of Medications		-1.83	0.39	< .0001

At the end of the 8-week intensive training, *health habits* among the intervention group increased by 3.1 units (SE = 1.0; effect size = 0.15) compared to the control group. This difference was sustained at 20 weeks, although at a lower level (2.4 units, SE = 0.2). The *p*-value for the omnibus group-by-time interaction effect was 0.0005. The main effect for group, interpretable as the difference between the two groups at baseline, was insignificant, as anticipated due to randomization.

**Fig. 3:**
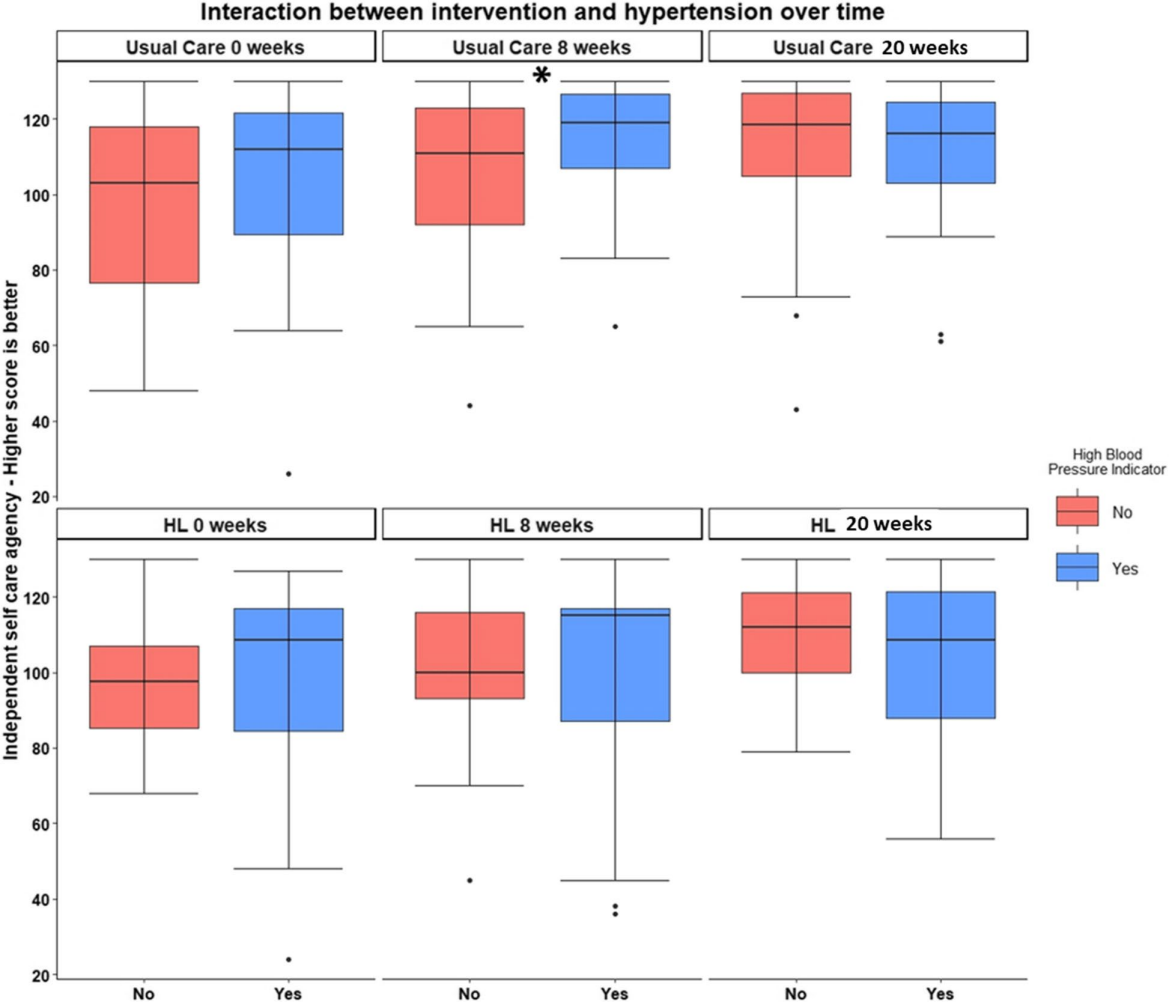
Graph of independent self-care agency showing a statistically significant interaction between high blood pressure, intervention, and time. The bars are the minimum/maximum points that fall within quartile 1–1.5*interquartile range and q3 + 1.5*IQR respectively, and the dots are extreme outliers (as defined by falling outside that range). *p = .0005

In addition, the independent self-care agency showed a statistically significant interaction between high blood pressure, intervention, and time. At eight weeks, the score for individuals with high blood pressure (HBP) and in the intervention group had a score, on average, 13.5 points higher than the control group without HBP (SE = 4.37, *p* = 0.0023; effect size = 0.3). However, that difference was not significant at 20 weeks (*p* = 0.47). Graphic display of this effect is shown in Fig. 3. The *p*-value of the omnibus group by time by blood pressure interaction effect was 0.007. Again, the main effect for the group was insignificant and not included in our model because our randomization was successful.

### Participant satisfaction

**Fig. 4:**
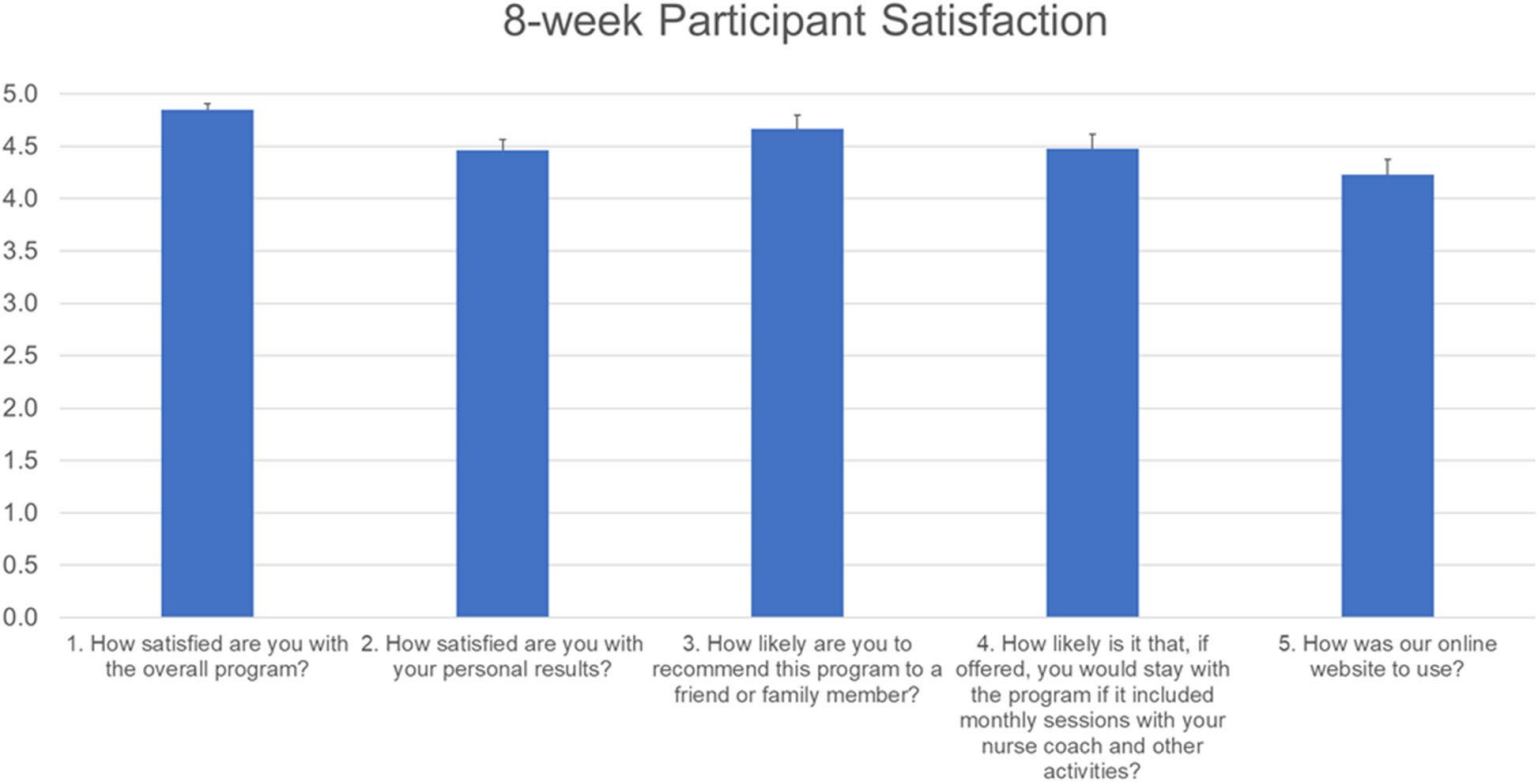
Mean response of HealthyLifetime participants to a five question satisfaction survey using a 6-point scale ranging from 0-Not at all satisfied/recommend/likely/easy to use to 5-Highly satisfied/recommend/likely/(Very easy to use)

At the end of the 8-week intervention, participants reported being well-satisfied with the HL program, including using the website, the launch site for all data collection, and videoconferencing (Fig. 4). On a six-point scale of 0 (lowest) to 5 (highest), only 13 of 122 participants entered responses of 2 or below. Of the 13 low-scoring participants, 10 were from the ‘usual care’ group, which may represent dissatisfaction in these few with being in the control condition, which did not include videoconferencing with the nurse.

## Discussion

### Principal results

The race, gender, and partner characteristics of our sample are not surprising as it has been reported that there is much higher participation in community centers and organizations by females than males [34, 35] and that there is a shrinking rate of coupled relationships among people over 50 [36–38]. African-American men also continue to have substantially lower life expectancy than African-American women, White women, and White men [39]. The recruitment from the HBEC essentially yielded African-American women.

A graphic display of outcome variables (Fig. 2) indicates improvement trends over time for *both* the HL and “usual care” groups for functional impairment, independent self-care agency, goal-setting confidence, and confidence in medication taking. However, regression analyses showed no significant benefit of the HL program for these outcomes versus the “usual care” condition, possibly because the latter included an opportunity to self-evaluate health through the PHS and health goal setting throughout the treatment and follow-up phases of the study. However, when we considered the influence of the dominant chronic disease in the model, which for the sample was HBP, the HBP subgroup demonstrated more significant improvement in the independent self-care agency at eight weeks than the “usual care” group without HBP. This effect for the HL group was not seen at 20 weeks. A similar outcome pattern was seen in an RCT of weekly health coaching for six months in Type 2 Diabetes Mellitus participants focused on increasing exercise and diet to reduce carbohydrate intake [35]. The intervention group achieved an accelerated hemoglobin A1c (HbA1c) reduction, leading to a significant between-group difference at three months; however, with no significant between-group differences in change of HbA1c at six months as the control group continued to improve with usual care.

As measured in this study, independent self-care agency relates to confidence in performing life activities independently, such as shopping, housework, and transportation, and managing aspects of chronic disease such as symptom “usual care” and medications. Since the question set was designed to assess self-efficacy in managing a chronic illness, it is a more relevant assessment for the subgroup of people with a similar chronic disease. Our nurse coaching HL intervention focused on addressing and improving participants’ confidence in managing their chronic condition and overall health; therefore, this improvement in self-efficacy for those with HBP is a promising benefit of the HL nurse coaching intervention.

Although the individual variables of activity, eating, and drinking showed improved trends over time, with higher trend values for the HL group, the individual regression results were insignificant. In contrast, the interaction of time and group effect of the composite variable outcome *health habits* was substantial. Given that the individual variable scales each had relatively few items, the composite variable *health habits*, which includes the total items among these variables, is a more robust measure of change as the number of items in a scale improves reliability when those items measure the same construct [40]. The items comprising *health habits* directly align with the top two priority goals selected by the HL intervention and “usual care” groups related to eating, weight loss, and exercise/activity, as displayed in Table 3. This may relate to the split priorities of participants between the focus on behaviors associated with changing food and alcohol consumption and behaviors to change exercise/activity, reducing the overall participant effort and program’s effect for each.

In recent years, there has been a shift from addressing individual risk factors/behaviors toward addressing multiple risk factors such as smoking, body weight, alcohol consumption, and exercise/activity. A recently published large-scale population-based cohort study found that the lowest risk of all-cause cardiovascular or cancer-related mortality across physical activity and diet combinations is achieved with the blend of the highest levels of self-reported physical activity and highest diet quality score [41]. Like our outcome measures, physical activity was hours/week, and diet quality was food types consumed rather than calories or biometric measurements such as body mass index. Our results indicate that measuring the total effect across several discrete risks provided the best evidence of participant effort and progress toward overall risk reduction and are commensurate with the best practice of incorporating multiple health behavior change interventions to maximize impact and cost-effectiveness [9].

Our HL program is delivered via videoconferencing technology (Zoom.com) with face-to-face nurse coaching, short-term (8 weeks), and intensive (weekly sessions 30–90 min) using specifically defined cognitive behavioral approaches in older adults with a range of chronic conditions. While others have shown more moderate satisfaction scores with the introduction of videoconferencing for clinical consultation with patients [42, 43], our participant satisfaction rates were high across all five dimensions (Fig. 4). Noteworthy is the high satisfaction scores for nurse interaction. The two-way videoconferencing enhanced this, allowing ‘face-to-face’ communication, which may have boosted satisfaction with the technology [44].

Moreover, we strove to standardize coaching methods and emulate the best health coaching practices. While our outcomes were designed with the anticipated functional decline of an aging population, the outcomes we demonstrated aligned with participants’ self-identified goals regardless of health or functional status. The finding for the HBP subgroup is similar to that of others [45] who found an accelerated improvement in the intervention group that was not observable at follow-up when control group outcomes matched the intervention group.

### Limitations

We used a ‘Risk of Bias’ assessment tool to retrospectively evaluate our study [46]. There were no deviations from the study protocol during implementation that could have introduced bias. Our final sample size rendered the study slightly underpowered from the preliminary estimate. Our recruitment methods produced a sample of high-functioning older adults, predominantly a race-balanced group of women with one or more chronic conditions. This limits generalizability to gender-diverse and more impaired populations. We also cannot conclude from this study the influence of the functional capacity of older adults on the ease of computer and video conferencing use – a relationship observed by others [47]. An inclusion criterion for our study was having access to a computer and internet connectivity capable of video streaming to allow high-fidelity video conferencing. Thus, our inclusion criterion confounds the relationship to functional ability, for even our low-functioning participants met this computer/internet criterion. There is also the inevitable potential of participant response bias due to treatment expectations, as the group assignments were not blinded. Yet we found high satisfaction with the treatment phase among most participants regardless of group assignment, suggesting participants had similar treatment expectations. These limitations are the natural consequence of a pragmatic pilot trial such as ours, and more research is needed to replicate our results in a larger sample.

## Conclusions

Our primary conclusion is that a nurse health coaching program had essential health-related lifestyle benefits over and above usual care in a higher-functioning sample of community-based older adults with chronic diseases. This is an important target population for primary care practices as the goal is to intervene early in aging to stave off the complications of chronic disease and functional decline. Our sustained improvement in *health habits* is important because changing health habits alone has been shown to reduce all-cause morbidity and mortality in chronic diseases [3]. While we and others have shown that short-term benefits can be achieved in outcomes directly related to disease/condition, such as HbA1c or our outcome of independent self-care agency in HBP participants, the longer view on changing overall health habits for multifactor risk reduction may be the most critical target outcome regardless of disease or condition.

Further, our program uses specific theory-based strategies and methods that are replicable, unlike many previous studies in health coaching, and can be tested in other populations. It is delivered through accessible electronic methods with high participant satisfaction. Preserving the face-to-face component with videoconference technology may be most meaningful to older adults [48].

Future studies should continue to address the standardization of videoconferencing health coaching methods, incorporating best practices with experimental designs that address ‘dose–response’ in target populations. A greater understanding of the relationship between the underlying mechanisms of effective behavior change, such as the role of participant-driven goal setting and the face-to-face component, to the complexities of multifactorial outcome measurement is required to predict better the efficacy and service ‘dose’ of health coaching in specified populations.

### Supplementary Information

**Additional file 1.**

## Data Availability

De-identified data from this study will be made available at the CT.gov registration site, record NCT05070923.

## Abbreviations

BCT: Behavior change techniques
HbA1c: Hemoglobin A1c
HBEC: Healthier Black Elders Center
HBP: High blood pressure
HL: Healthy Lifetime
IADL: Instrumental Activities of Daily Living
IRB: Institutional Review Board
MI: Motivational Interviewing
PHS: Personal Health Survey
RCT: Randomized controlled trial
SD: Standard Deviation
SE: Standard Error
SMRC: Self-Management Resource Center of Stanford University
