# Supplementary material for: A randomized trial of a theory-driven model of health coaching for older adults: short-term and sustained outcomes
Source: BMC Prim Care. 2023 Oct 5;24:205. doi: 10.1186/s12875-023-02162-x (PMC10552322; doi:10.1186/s12875-023-02162-x)
Supplement: Supplementary file 1 — Additional file 1. [file 12875_2023_2162_MOESM1_ESM.docx]

HL Personal Health Survey

Participant ID


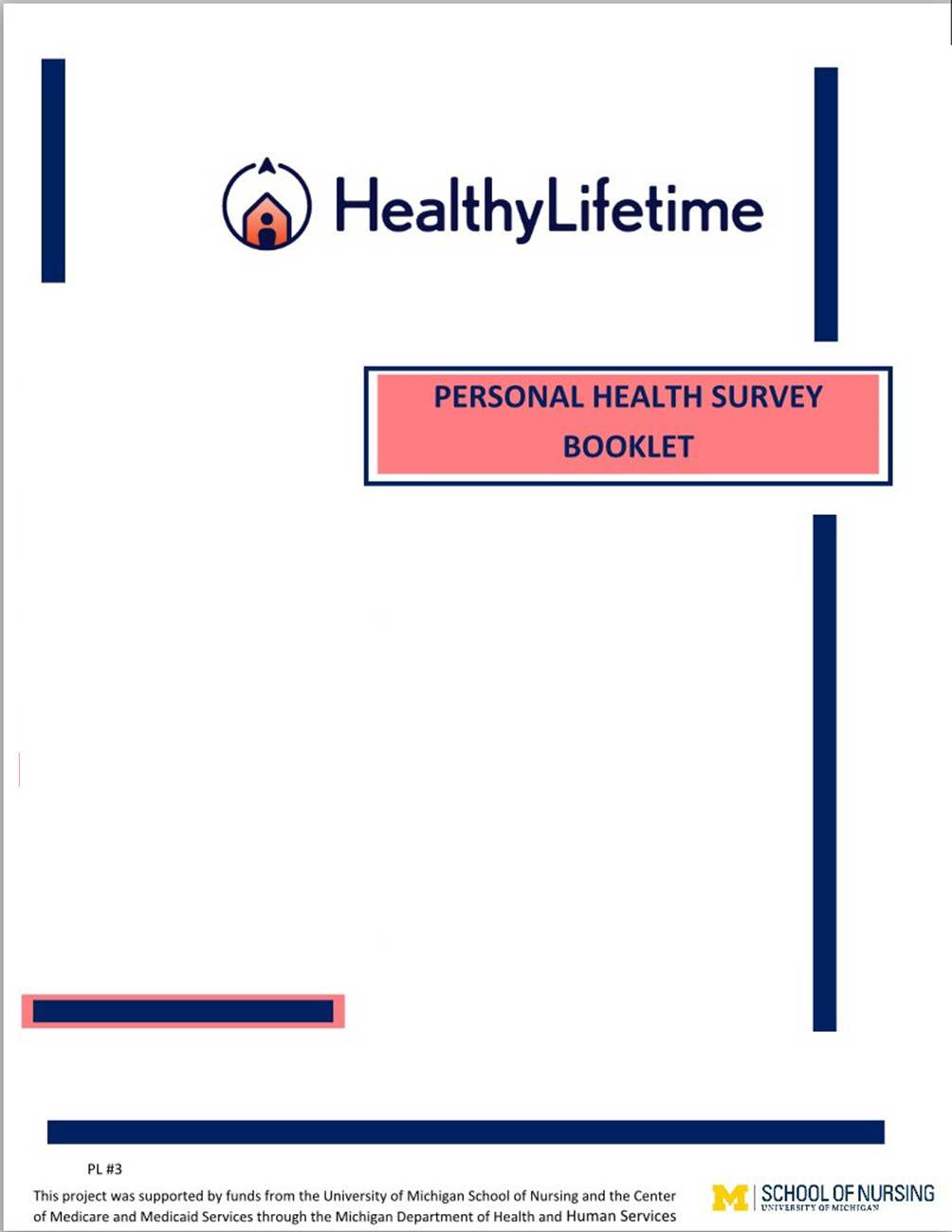


# Personal Health Survey

**This is a *Personal Health Survey* which will provide information to guide us in helping you better manage your health care while living at home. Please answer the questions as best as you can; there are no ‘right’ or ‘wrong’ answers.**

**You may feel that some of the questions or choice of answers do not directly apply to you, please still choose an answer that you think fits best.**

**Read and answer each question one at a time as instructed in the survey. Please note there are differences in the questions, the scales, and the time periods asked about throughout this survey.**

**If you need to stop and take a break, you may do so but please save your work so that you can just pick up where you left off when you come back.**

# BACKGROUND INFORMATION

1. **In what year were you born? (Enter 4-digit birth year, for example, 1976):***
2. **Gender: (CHECK ONE BOX):***


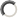

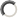
*Please select one* Male Female


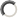
Prefer not to answer

1. **Marital Status (CHECK ONE BOX BELOW):***


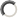

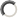
*Please select one* Married Partnered


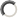

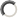
Single, Never Married Separated


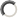
Divorced


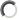
Widowed

1. **Race or Ethnic Origin (CHECK ONE BOX BELOW):***

*Please select one*


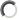

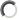
White or Caucasian Asian


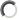

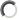
Arab American/Middle Eastern More than one race


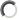

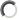

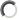
Black or African American American Indian or Alaska Native Hispanic or Latino


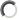

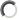
Native Hawaiian/Other Pacific Islander Unknown

1. **What is your highest level of education? (CHECK ONE BOX BELOW):***

*Please select one*


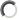
Grade school


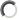

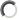
Some high school (1-3 years) High School Diploma


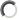

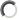
Trade/Technical/Vocational Training Some college


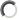
Bachelor’s Degree


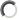
Graduate or Professional School

1. **What is your current work status? (CHECK ALL THAT APPLY)***

*Please select all that apply*


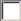
Employed for wages


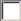
Out of work and looking for work


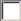

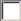
Out of work but not currently looking for work Homemaker


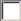

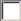
Retired Disabled

1. **What is your total household income? (CHECK ONE BOX BELOW)***

*Please select one*


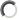
$0 – $20,000


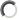
$21,000 - $50,000


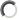
$51,000 - $100,000


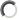
Greater than $100,000

# SOCIAL AND SPIRITUAL NETWORK AND SUPPORT

FOR EACH OF THE FOLLOWING QUESTIONS, CHECK THE NUMBER THAT BEST REPRESENTS YOU OR YOUR SITUATION ***AT THE PRESENT TIME***:

1. **Spiritual beliefs and practice give me hope or comfort. ***


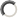

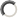
*Please select one* 1 - Not at all 2


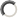
3


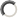
4


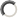
5 - Always

1. **People I know will help me when I need it.***


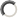

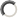
*Please select one* 1 - Not at all 2


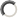
3


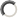
4


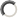
5 - Always

1. **I receive care services from others (e.g. help with personal care, managing my bills, doing chores).***


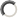

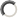
*Please select one* 1 - Not at all 2


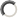
3


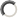
4


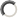
5 - Always

1. **I engage in social activities with family, friends, neighbors and groups.***


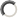

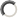
*Please select one* 1 - Not at all 2


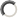
3


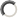
4


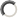
5 - Always

1. **I provide care services to others.***


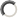

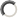
*Please select one* 1 - Not at all 2


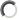
3


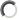
4


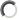
5 - Always

1. **I have adequate means of transportation to/from routine activities (e.g. grocery shopping, church services).***


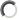

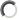
*Please select one* 1 - Not at all 2


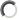
3


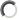
4


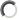
5 - Always

1. **My current income meets my needs.***


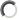

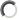
*Please select one* 1 - Not at all 2


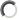
3


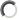
4


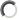
5 - Always

1. **I am satisfied with my sexual activity.**


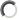

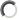
*Please select one* 1 - Not at all 2


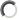
3


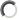
4


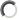
5 - Always

1. **I am well rested when I get up from sleep.***


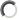

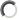
*Please select one* 1 - Not at all 2


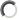
3


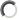
4


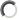
5 - Always

# HEALTH STATUS INDICATORS

FOR EACH OF THE FOLLOWING QUESTIONS, CHECK THE NUMBER THAT BEST REPRESENTS YOU OR YOUR

SITUATION ***AT THE PRESENT TIME***:

1. **In the past 3 months, how many times have you had an unplanned medical clinic visit with your doctor or primary medical provider? (Unplanned means that the visit was not a routine check-up or a planned check-up for your ongoing health problems.) (CHECK ONE BOX BELOW)***

*Please select one*


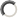
0


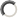
1


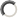
2


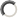
3


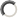
4


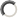
5


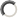
6


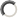
7 or more

1. **In the past 3 months, how many times have you had to go to the Emergency Room for a medical problem?” (CHECK ONE BOX BELOW)***

*Please select one*


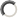
0


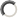
1


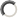
2 or more

1. **In the past 3 months, how many times have you had an overnight stay in the hospital? (CHECK ONE BOX BELOW)***

*Please select one*


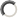
0


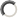
1


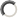
2 or more

1. **Which, if any, of the following major changes have you had in your life recently? (CHECK ALL THAT APPLY)***

*Please select all that apply*


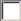

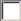
Death of someone close to you Major illness


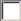

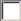
Money or Income Problems Divorce/Separation


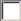
Moved to another home Other

None/No recent major changes in my life

# PERSONAL HEALTH RATING

1. **In general, would you say your health RIGHT NOW is: (CHECK ONE CIRCLE BELOW) ***

*Please select one*

1. - Excellent
2. - Very Good
3. - Good
4. - Fair
5. - Poor

In the next questions we ask you to indicate how you expect your health to change in the next 3 years.

1. **In general, I think my health in THREE YEARS will be… (CHECK ONE RESPONSE BELOW)***

*Please select one*

1. - Excellent
2. - Very Good 2 - Good
3. - Fair
4. - Poor

# LIFESTYLE HABITS

***Tobacco and Alcohol***

1. **Do you currently smoke or use tobacco?***

*Please select one*

Yes No

1. **Do you drink alcohol? ***

*Please select one*

Yes > CONTINUE TO Q.25

No -----> SKIP TO Q.26

1. **How many total mixed drinks, beers, and glasses of wine do you consume in a week? (CHECK THE BOX BELOW INDICATING THE TOTAL NUMBER OF MIXED DRINKS, BEERS, AND/OR GLASSES OF WINE YOU DRINK IN A WEEK) ***

*Please select one*

<1

1

2

3

4

5

6

7

8 or more

1. **Do you use any type of marijuana?***

*Please select one*

Yes No

# Exercise and Activities

**DURING THE PAST WEEK**, even if it was not a typical week for you, how much total time (*for the* ***entire week***) did you spend on each of the following?

# CHECK ONE RESPONSE FOR EACH EXERCISE/ACTIVITY

1. **Stretching and strengthening exercises (range of motion, using weights, yoga, Pilates, Tai chi, etc.)***

*Please select one*

None

Less than 30 Minutes/Week 30-60 minutes/week

1-3 hours/week

More than 3 hours/week

1. **Walking***

*Please select one*

None

Less than 30 Minutes/Week

30-60 minutes/week

1-3 hours/week

More than 3 hours/week

1. **Swimming or water exercise***

*Please select one*

None

Less than 30 Minutes/Week 30-60 minutes/week

1-3 hours/week

More than 3 hours/week

1. **Bicycling (including stationary exercise bikes and treadmill)***

*Please select one*

None

Less than 30 Minutes/Week 30-60 minutes/week

1-3 hours/week

More than 3 hours/week

1. **Other exercise***

*Please select one*

None

Less than 30 Minutes/Week 30-60 minutes/week

1-3 hours/week

More than 3 hours/week

# FOOD CONSUMPTION

**Please CHECK the best response** that tells the ***frequency*** of eating each food group **in the last four weeks.**

1. **Vegetables (such as Dark green vegetables, red and orange vegetables, starchy vegetables, beans and peas)***

*Please select one* Seldom or Never Once a Week

1. or 2 Times a Week

Once a Day

1. or More times a Day
2. **Fruits excluding juices (such as Apples, bananas, oranges, grapes, tangerines, cherries, etc.)***

*Please select one* Seldom or Never Once a Week

1. or 2 Times a Week Once a Day
2. or More times a Day
3. **Whole grains (such as brown rice, whole grain bread and cereal, whole grain pasta)***

*Please select one* Seldom or Never Once a Week

1. or 2 Times a Week Once a Day
2. or More times a Day
3. **Refined Grains (such as white rice, white bread, white pastas)***

*Please select one* Seldom or Never Once a Week

1. or 2 Times a Week Once a Day
2. or More times a Day
3. **Dairy (such as milk, yogurt, cottage cheese, cheese)***

*Please select one* Seldom or Never Once a Week

1. or 2 Times a Week Once a Day
2. or More times a Day
3. **Non-dairy alternatives (such as almond milk, coconut milk, soymilk, non-dairy spreads)***

*Please select one* Seldom or Never Once a Week

1. or 2 Times a Week Once a Day
2. or More times a Day
3. **Protein foods, Meat and Seafood (such as beef, pork, chicken, soy and whey protein products, fish)***

*Please select one* Seldom or Never Once a Week

1. or 2 Times a Week Once a Day
2. or More times a Day
3. **Oils (such as Olive oil, cooking oils, oil-based salad dressings)***

*Please select one* Seldom or Never Once a Week

1. or 2 Times a Week Once a Day
2. or More times a Day
3. **Sugary food (such as candy, cookies, cake) and drinks (soda, juice)***

*Please select one* Seldom or Never Once a Week

1. or 2 Times a Week Once a Day
2. or More times a Day

# HEALTH IMPACT

**CHECK ONE NUMBER FOR EACH QUESTION BELOW**

***AT THE PRESENT TIME…***

1. **Is your health interfering with your normal social activities with family, friends, neighbors or groups?**

*****

*Please select one*

1. - Not at All
2. - Slightly
3. - Moderately
4. - Quite a bit
5. - Almost Totally
6. **Is your health interfering with your normal hobbies or recreational activities?***

*Please select one*

1. - Not at All
2. - Slightly
3. - Moderately
4. - Quite a bit
5. - Almost Totally
6. **Is your health interfering with doing your household chores?***

*Please select one*

1. - Not at All
2. - Slightly
3. - Moderately
4. - Quite a bit
5. - Almost Totally
6. **Is your health interfering with your errands and shopping?***

*Please select one*

1. - Not at All
2. - Slightly
3. - Moderately
4. - Quite a bit
5. - Almost Totally

# SYMPTOM INVENTORY

How often do you experience the following symptoms?

CHECK ONE NUMBER FOR EACH SYMPTOM BELOW

1. **Physical Discomfort***

*Please select one*

1. - Never
2. - Almost Never
3. - Sometimes
4. - Fairly Often
5. - Very Often
6. - Always
7. **Pain**

*Please select one*

1. - Never
2. - Almost Never
3. - Sometimes
4. - Fairly Often
5. - Very Often
6. - Always
7. **Tiredness or fatigue***

*Please select one*

1. - Never
2. - Almost Never
3. - Sometimes
4. - Fairly Often
5. - Very Often
6. - Always
7. **Shortness of Breath***

*Please select one*

1. - Never
2. - Almost Never
3. - Sometimes
4. - Fairly Often
5. - Very Often
6. - Always
7. **Feeling 'blue' or sad***

*Please select one*

1. - Never
2. - Almost Never
3. - Sometimes
4. - Fairly Often
5. - Very Often
6. - Always
7. **Feeling anxious or worried***

*Please select one*

1. - Never
2. - Almost Never
3. - Sometimes
4. - Fairly Often
5. - Very Often
6. - Always

Over the **PAST TWO WEEKS**, how often have you been bothered by the following problems?

CHECK ONE NUMBER FOR EACH SYMPTOM BELOW

1. **Little interest or pleasure in doing things?***

*Please select one*

1. - Not at All
2. - Several Days
3. - More than Half the Days
4. - Nearly Every Day
5. **Feeling down, depressed, or hopeless?***

*Please select one*

1. - Not at All
2. - Several Days
3. - More than Half the Days
4. - Nearly Every Day

# HOUSEHOLD ACTIVITIES

**AT THE PRESENT TIME**, for each of the following daily activities, please**CHECK THE ONE RESPONSE** for each activity that best describes your ability to do the activity.

Daily Living Activity

1. **Shopping***

*Please select one* 1 - Cannot Do 2

3

4

1. - Do Independently
2. **Cooking***

*Please select one* 1 - Cannot Do 2

3

4

5 - Do Independently

1. **Managing medications***

*Please select one* 1 - Cannot Do 2

3

4

5 - Do Independently

1. **Using the phone***

*Please select one* 1 - Cannot Do 2

3

4

5 - Do Independently

1. **Doing housework***

*Please select one* 1 - Cannot Do 2

3

4

5 - Do Independently

1. **Doing laundry***

*Please select one* 1 - Cannot Do 2

3

4

5 - Do Independently

1. **Driving or using public Transportation ***

*Please select one* 1 - Cannot Do 2

3

4

5 - Do Independently

1. **Managing finances***

*Please select one* 1 - Cannot Do 2

3

4

5 - Do Independently

# CONFIDENCE IN DOING ACTIVITIES

We would like to know how confident you are in doing certain activities. For the purposes of answering these questions, we define CONFIDENCE as the belief in your chances of being able to do and/or complete this activity or task successfully, however you define that.

For example:

You are shown an object that weighs 5 pounds and are asked if you can pick it up. You*know* that you can pick up that 5-pound weight. For our purposes, you are ***confident or certain*** that you can pick up the five-pound weight.

For each of the following questions, please**CHECK THE NUMBER** that corresponds to your confidence or certainty that you can do the activity or task **AT THE PRESENT TIME:**

1. **How confident are you that you can continue to do your hobbies and recreation?***

*Please select one*

1 - Not at all Confident 2

3

4

5

6

7

8

9

10 - Totally confident)

1. **How confident are you that you can continue to do the things you like to do with friends and family (such as social visits and recreation)?***

*Please select one*

1 - Not at all Confident 2

3

4

5

6

7

8

9

10 - Totally confident)

1. **How confident are you that you will be able to continue to do your household chores?***

*Please select one*

1 - Not at all Confident 2

3

4

5

6

7

8

9

10 - Totally confident)

1. **How confident are you that you will be able to continue to do errands and shopping?***

*Please select one*

1 - Not at all Confident 2

3

4

5

6

7

8

9

10 - Totally confident)

# CONFIDENCE IN MANAGING YOUR SYMPTOMS

For each of the following questions, please**CHECK THE NUMBER** that corresponds to your confidence or certainty that you can do the activity or task **AT THE PRESENT TIME:**

1. **How confident are you that you can keep fatigue from interfering with the things you want to do?***

*Please select one*

1 - Not at all Confident 2

3

4

5

6

7

8

9

10 - Totally confident)

1. **How confident are you that you can keep the physical discomfort or pain from interfering with the things you want to do?***

*Please select one*

1 - Not at all Confident 2

3

4

5

6

7

8

9

10 - Totally confident)

1. **How confident are you that you can keep the emotional distress from interfering with the things you want to do?***

*Please select one*

1 - Not at all Confident 2

3

4

5

6

7

8

9

10 - Totally confident)

1. **How confident are you that you can keep the any other symptoms or health problems you have from interfering with the things you want to do?***

*Please select one*

1 - Not at all Confident 2

3

4

5

6

7

8

9

10 - Totally confident)

1. **How confident are you that you can manage your health condition so as to reduce your need to see a doctor?***

*Please select one*

1 - Not at all Confident 2

3

4

5

6

7

8

9

10 - Totally confident)

1. **How confident are you that you can do things other than just taking medication to reduce how much your illness affects your everyday life?***

*Please select one*

1 - Not at all Confident 2

3

4

5

6

7

8

9

10 - Totally confident)

1. **How confident are you that you will be able to stick with the behavior changes you are making?***

*Please select one*

1 - Not at all Confident 2

3

4

5

6

7

8

9

10 - Totally confident)

1. **How confident are you that you will be successful in meeting your goals?***

*Please select one*

1 - Not at all Confident 2

3

4

5

6

7

8

9

10 - Totally confident)

1. **How confident are you that your efforts will improve your health?***

*Please select one*

1 - Not at all Confident 2

3

4

5

6

7

8

9

10 - Totally confident)

# CONFIDENCE IN MEDICATION TAKING

For the following questions include **ALL** medications – prescriptions from your doctor; over-the-counter drugs such as aspirin or pain relievers; homeopathic medicines; vitamins, minerals and herbs; natural substances such as you might get at a ‘health food store’; other health products.

CHECK ONE NUMBER for each statement below.

1. **How confident are you that you take your medications correctly… if you take several different medicines each day?***

*Please select one*

1. - Not at all Confident
2. - Somewhat Confident
3. - Very Confident
4. **How confident are you that you take your medications correctly… if you take medicines more than once a day?***

*Please select one*

1. - Not at all Confident
2. - Somewhat Confident
3. - Very Confident
4. **How confident are you that you take your medications correctly… if you are away from home?***

*Please select one*

1. - Not at all Confident
2. - Somewhat Confident
3. - Very Confident
4. **How confident are you that you take your medications correctly… if you have a busy day planned?***

*Please select one*

1. - Not at all Confident
2. - Somewhat Confident
3. - Very Confident
4. **How confident are you that you take your medications correctly… if they cause some side effects?***

*Please select one*

1. - Not at all Confident
2. - Somewhat Confident
3. - Very Confident
4. **How confident are you that you take your medications correctly… if no one reminds you to take the medicine?***

*Please select one*

1. - Not at all Confident
2. - Somewhat Confident
3. - Very Confident
4. **How confident are you that you take your medications correctly… if the schedule to take the medicine is not convenient?***

*Please select one*

1. - Not at all Confident
2. - Somewhat Confident
3. - Very Confident
4. **How confident are you that you take your medications correctly… if your normal routine gets messed up?***

*Please select one*

1. - Not at all Confident
2. - Somewhat Confident
3. - Very Confident
4. **How confident are you that you take your medications correctly… if you are not sure how to take the medicine?***

*Please select one*

1. - Not at all Confident
2. - Somewhat Confident
3. - Very Confident
4. **How confident are you that you take your medications correctly… if you are not sure what time of the day to take your medicine?***

*Please select one*

1. - Not at all Confident
2. - Somewhat Confident
3. - Very Confident
4. **How confident are you that you take your medications correctly… if you are feeling sick (you know, like having a cold or the flu)?***

*Please select one*

1. - Not at all Confident
2. - Somewhat Confident
3. - Very Confident
4. **How confident are you that you take your medications correctly… if you are not sure how it works or what it does for you?***

*Please select one*

1. - Not at all Confident
2. - Somewhat Confident
3. - Very Confident
4. **How confident are you that you will be able to afford your medicines?***

*Please select one*

1. - Not at all Confident
2. - Somewhat Confident
3. - Very Confident
4. **How confident are you that you will be able to get to the pharmacy to get your medicines?***

*Please select one*

1. - Not at all Confident
2. - Somewhat Confident
3. - Very Confident

# TOP HEALTH ISSUES AND GOALS FOR BETTER HEALTH

1. **What are your top health issues? PLEASE TYPE IN THE BOX BELOW:***
2. **Are you working on achieving any health goals right now? Examples may be things like: eating more fruits and vegetables, exercising at least 3 times a week for 30 minutes, getting at least 7 hours of sleep a night, taking my medicines as directed….or anything else you think important to your health.***

*Please select one*

Yes > CONTINUE TO Q.90

No -----> SKIP TO Q.99

If you are working on health goals, please list the top goal or goals [up to a total of three] you are working on AND answer two questions about the goal or goals:

1. **Goal #1 is: (TYPE IN BOX BELOW)**
2. **Please rate this goal in overall importance to you with ‘0’ meaning ‘not important at all now’ and ’10’ meaning ‘highest importance now’ (CHECK ONE NUMBER BELOW):***

*Please select one*

0 - Not Important at all Now 1

2

3

4

5

6

7

8

9

10 - Highest Importance Now

1. **Please rate how confident you are in achieving this goal right now where ‘0’ means ‘not confident at all’ and ’10’ means ‘completely confident’ (CHECK ONE NUMBER BELOW):**

*Please select one*

0 - Not Confident at all 1

2

3

4

5

6

7

8

9

10 - Completely Confident

1. **Goal #2 is: (TYPE IN BOX BELOW)**
2. **Please rate this goal in overall importance to you with ‘0’ meaning ‘not important at all now’ and ’10’ meaning ‘highest importance now’ (CHECK ONE NUMBER BELOW ):**

*Please select one*

0 - Not Important at all Now 1

2

3

4

5

6

7

8

9

10 - Highest Importance Now

1. **Please rate how confident you are in achieving this goal right now where ‘0’ means ‘not confident at all’ and ’10’ means ‘completely confident’ (CHECK ONE NUMBER BELOW):**

*Please select one*

0 - Not Confident at all 1

2

3

4

5

6

7

8

9

10 - Completely Confident

1. **Goal #3 is: (TYPE IN BOX BELOW)**
2. **Please rate this goal in overall importance to you with ‘0’ meaning ‘not important at all now’ and ’10’ meaning ‘highest importance now’ (CHECK ONE NUMBER BELOW):**

*Please select one*

0 - Not Important at all Now 1

2

3

4

5

6

7

8

9

10 - Highest Importance Now

1. **Please rate how confident you are in achieving this goal right now where ‘0’ means ‘not confident at all’ and ’10’ means ‘completely confident’ (CHECK ONE NUMBER BELOW):**

*Please select one*

0 - Not Confident at all 1

2

3

4

5

6

7

8

9

10 - Completely Confident

1. **Please list any other issues or concerns you may want us to know about below: (TYPE IN BOX BELOW)**

THANK YOU FOR COMPLETING YOUR PERSONAL HEALTH SURVEY!
